# Causal or not: applying the Bradford Hill aspects of evidence to the association between Zika virus and microcephaly

**DOI:** 10.15252/emmm.201506058

**Published:** 2016-03-14

**Authors:** Christina Frank, Mirko Faber, Klaus Stark

**Affiliations:** ^1^ Department for Infectious Disease EpidemiologyRobert Koch InstituteBerlinGermany

**Keywords:** Microbiology, Virology & Host Pathogen Interaction

## Abstract

While experts have suspected a causal link between outbreaks of Zika virus and microcephaly, it was not demonstrated. The currently available data are here organized into the Austin Bradford Hill's aspects of evidence for the consideration of causality.

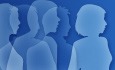

After the emergence of unusual clusters of microcephaly among babies born in the fall of 2015 in Brazil—and, retrospectively, in French Polynesia—WHO declared a Public Health Emergency of International Concern in January 2016 (http://www.who.int/mediacentre/news/statements/2016/1st-emergency-committee-zika/en/). Based on the spatial and temporal correlation of these clusters with outbreaks of Zika virus infections a few months before, a causal link is suspected. Whether the observed link between infection and microcephaly is indeed causal needs careful assessment—not the least because we might fall into the trap of ecological fallacy, inferring a causal association on the individual level from an association on the aggregate or population level.

Here, we organize the currently available data into Austin Bradford Hill's aspects of evidence for the consideration of causality (Hill, 1965). Although it is no longer considered a litmus test in itself, Hill's recipe for careful and multifaceted contemplation of the available evidence is still a rigorous method for separating what is known from what is not known. For each aspect, we first consider individual‐level evidence, but supplement it with likely more readily observable effects on the population level.

## Strength of the association

Studies to characterize the strength of this association in terms of odds ratios from case–control studies or (preferable) relative risks from cohort studies are already underway. These should be able to answer how much more likely a mother infected with Zika virus any time during pregnancy, or in a specific time window, will be giving birth to a child with microcephaly compared to mothers who were not infected. On the ecological level, a strong increase in microcephaly cases is not by itself evidence for a strong causal association because there might be confounding or biases. Nevertheless, if there were a strong causal association, we would expect to see a large increase in cases of microcephaly in a population with many Zika virus infections during pregnancy.

## Consistency

If the association is causal, maternal Zika virus infection would consistently predict a risk of microcephaly in the fetus or newborn. But such data are not available yet. Isolated cases of congenital microcephaly and Zika virus infection outside the outbreak areas, such as the travel‐associated cases on Hawaii and in Slovenia, provide some limited evidence toward a consistent association regardless of area of permanent residence (Mlakar *et al*, 2016).

On the ecological level, the incidence of microcephaly apparently increased in temporal and spatial association with virus outbreaks in Brazil and French Polynesia—two out of two countries with larger populations, for which the outcomes of early pregnancies potentially affected during the virus outbreak are already known. However, this is not yet the case in many other countries in the Americas that experienced Zika virus outbreaks in recent months. Thus, the current lack of microcephaly clusters there does not argue against causality.

On first glance, it is inconsistent and even a bit irritating that outbreaks of microcephaly have not been observed in other parts of the world that likely are endemic for Zika virus, namely tropical areas in Africa and Asia. Potential reasons include high infant mortality, and patchy perinatal care and surveillance systems. In addition, endemic Zika virus infection could generate high levels of immunity within the population: If most people were infected during childhood, women of child‐bearing age would be largely immune and congenital infections rare. To ascertain the status of infection and immunity in endemic regions requires serosurveys using diagnostic tools that can reliably differentiate between antibodies to the various flaviviruses, especially dengue virus. If the populations in tropical Africa and Asia are indeed largely immune, the association between Zika virus and microcephaly may only have come to light in Brazil, because the virus, uncommonly, met an immunologically naïve population of substantial size in a country with surveillance for congenital birth defects.

## Specificity

Specificity is always a problematic aspect for diseases that may have more than one cause. A causal relationship between Zika virus infection during pregnancy and microcephaly of the child cannot be specific, because other causes for microcephaly abound, including infections with cytomegalovirus, rubella virus, or *Toxoplasma gondii*. However, it may be possible to determine a certain type of impact on the developing brain that is specific to Zika virus. This would be important evidence for a causal association.

## Temporality

Many individual cases show the right order of Zika virus infection of the mother and microcephaly in the newborn. Detailed data on this temporal association will also help to identify the specific time window during pregnancy when the fetus' developing brain would be vulnerable to the virus. On the population level, we can see the same temporal pattern of microcephaly clusters following Zika virus outbreaks in French Polynesia and in Brazil. If temporality holds, we would expect an increase in congenital microcephaly in summer 2016 in the other countries that have been apparently affected by the virus since late 2015/early 2016.

## Biological gradient

Infection is a binary condition, and “dose–response”‐type evidence is therefore very rare in this context. However, there may be a graded association between the level of viremia in the mother or the severity of her symptoms and the probability that the child is born with microcephaly. Specific pathological data associated with Zika virus infection at certain phases during pregnancy—akin to “the earlier in pregnancy the mother is infected, the worse the grade of microcephaly”—could be interpreted as somewhat analogous evidence. On the population level, if the association is causal, we should see a stronger increase in the incidence of microcephaly where more pregnant women were potentially infected with Zika virus.

## Plausibility

A number of infectious agents are known to interfere with morphogenetic processes in the embryo or fetus during the blastogenesis period, resulting in congenital malformations owing to cytotoxic effects or inhibition of mitosis. In the case of Zika virus, various reports found viral RNA and antigens in amniotic fluid of infected mothers and the brains of microcephaly‐affected fetuses (e.g., Mlakar *et al*, 2016) and newborns who died after birth (e.g., Martines *et al*, 2016). These reports demonstrate congenital Zika virus infection, including penetration of the placenta and the fetal blood–brain barrier. Based on what is already known about the virus, it appears to have neuropathological properties (as discussed by Tetro, 2016), which would fit findings of fetal microcephaly such as in the comprehensively investigated case in Slovenia (Mlakar *et al*, 2016). Since the specific malformations appeared typical for an infectious cause, and since other infectious causes of microcephaly were actively ruled out, this individual case clearly points toward a causal role of Zika virus.

## Coherence

There is some, albeit seasoned, information that Zika is a neurotropic virus in experimental animals (Dick *et al*, 1952; Bell *et al*, 1971). Further studies in animal models are just beginning. If Zika virus infection in the gravid animal model would show associations with microcephaly and comparable changes of the central nervous system (CNS) in the offspring, or if matching neuropathological effects of the virus could be observed in cell culture, it would further support causality. Given that there are other viral infections already known to be causally associated with microcephaly and other changes of the CNS, adding Zika virus to the list would not violate any established scientific concepts.

## Experiment

Regarding the association between Zika virus and microcephaly, this aspect only pertains to data from animal models or “natural” experiments on the population level. In terms of the latter, it will be important and interesting to study differences in the association between virus infection and microcephaly between different population groups in Brazil—the poor and the affluent, or those who sought treatment and those who did not, or those with additional exposures or not. The role of previous exposure to other viruses and resulting effects on the immune system should also be studied. This will help to identify other, potentially relevant factors influencing the association between maternal Zika virus infection and congenital microcephaly. In addition, more cases similar to the Slovenian case—congenital microcephaly with evidence of prior maternal infection during brief periods of travel to outbreak areas—would argue against a role of potential (co‐)factors specific to being pregnant in Brazil and other affected areas.

## Analogy

There is some evidence that a number of other flaviviruses can cause congenital brain malformations in humans and animals. Among the animal pathogenic flaviviruses, Wesselsbron or Japanese encephalitis viruses are known to cause teratogenic effects (for details, see Tsai, 2006). During the large West Nile virus (WNV) epidemics in North America, one case of congenital infection with resulting CNS damage was reported (Alpert *et al*, 2003). But more extensive studies of infants whose mothers became infected during pregnancy did not demonstrate a large risk of congenital infection and “outbreaks” of brain malformations trailing the WNV outbreaks were not identified.

Causal links specific to microcephaly are established for viruses from other families, such as *Cytomegalovirus* or rubella virus. The analogy to rubella is particularly intriguing: Rubella virus is a *Togavirus* and maternal infections during pregnancy can cause congenital rubella syndrome (CRS) in the unborn child. Among the manifestations of CRS is microcephaly with cerebral calcifications (Katz *et al*, 1968), as has been described in many apparently Zika virus‐associated microcephaly cases (Mlakar *et al*, 2016). Before vaccines became available, rubella epidemics and even pandemics resulted in clusters of CRS whenever the virus met a large proportion of pregnant women who were not immune. In countries with rubella vaccination programs during childhood, where almost all women of childbearing age are immune against the virus, CRS has become very rare—which could be an interesting analogy to the apparent lack of clusters of microcephaly due to Zika virus in Africa and Asia.

Although the initial suspicion of a causal relationship was based on observed clusters of microcephaly trailing Zika virus outbreaks on the population level, we can already see other evidence supporting a causal relationship in regard to some of Austin Bradford Hill's aspects. This is particularly true for temporality, biological plausibility, and analogy. But clearly many gaps remain to be filled until we can decide “that the most likely explanation is causation” (Hill, 1965). Especially data from prospective or retrospective cohort studies in the affected countries are eagerly awaited to discern the strength of association (or lack thereof), and to address knowledge gaps for some of the other aspects. On the ecological level, it will be critical whether we will see clusters of microcephaly in the countries that were only affected in late 2015/early 2016, and whether the so far‐unexplained absence of microcephaly clusters in Africa and Asia can be elucidated.

This Opinion only addresses the putative link between Zika virus infection during pregnancy and congenital microcephaly. In addition, there are hints of associated ocular problems in newborns (Ventura *et al*, 2016), somewhat analogous to CRS. In French Polynesia, reports of microcephaly describe more variable congenital CNS malformations. Should causality between Zika virus and microcephaly be established, this phenotypic and comparatively observable characteristic may just be a proxy for a whole syndrome of congenital Zika virus infection, for which the same aspects of evidence ought to be checked. The question whether Zika virus can also cause Guillain–Barré syndrome as a post‐infectious sequel in non‐congenital infections should be considered separately.

Because we cannot yet characterize the association between Zika virus and microcephaly as causal, that in itself is no reason to shift the investigative focus to other hypotheses. Any other putative factors put forward as demonstrating similarly lagged correlations with recognized clusters of microcephaly, such as exposures to certain insecticides or medications, must be put through the same rigorous process of judging the evidence. They can be considered in case–control and cohort studies as additional exposures, to either confirm or refute any role in the causation of microcephaly in the groups of women who are studied.

We hope that applying the Bradford Hill aspects of evidence for epidemiological causation provides a useful checklist for current and future evidence for or against a causal association between Zika virus infection in pregnancy and congenital microcephaly. Giving the ongoing epidemiological and molecular research, especially in the Americas, some of the questions that we raised in this Opinion may already be answered by the time this article is published.
